# Omega-3 Fatty Acids Reduce Remnant-like Lipoprotein Cholesterol and Improve the Ankle–Brachial Index of Hemodialysis Patients with Dyslipidemia: A Pilot Study

**DOI:** 10.3390/medicina60010075

**Published:** 2023-12-30

**Authors:** Yusuke Kobayashi, Tetsuya Fujikawa, Aiko Haruna, Rina Kawano, Moe Ozawa, Tatsuya Haze, Shiro Komiya, Shota Suzuki, Yuki Ohki, Akira Fujiwara, Sanae Saka, Nobuhito Hirawa, Yoshiyuki Toya, Kouichi Tamura

**Affiliations:** 1 Center for Novel and Exploratory Clinical Trials (Y-NEXT), Yokohama City University, Yokohama 236-0004, Japan; 2 Department of Medical Science and Cardiorenal Medicine, Yokohama City University Graduate School of Medicine, Yokohama 236-0004, Japantamukou@yokohama-cu.ac.jp (K.T.); 3 Center for Health Service Sciences, Yokohama National University, Yokohama 240-8501, Japan; 4 Department of Nephrology and Hypertension, Yokohama City University Medical Center, Yokohama 232-0024, Japanhirawa@yokohama-cu.ac.jp (N.H.); 5 Department of Nephrology and Hypertension, Saiseikai Yokohamashi Nanbu Hospital, Yokohama 234-0054, Japan

**Keywords:** dyslipidemia, hemodialysis, omega-3 fatty acid, peripheral artery disease, remnant-like lipoprotein cholesterol

## Abstract

*Background and Objectives:* Omega-3 fatty acids have potent lipid-lowering and antiplatelet effects; however, randomized controlled trials have yet to examine the effect of high-dose omega-3 fatty acid administration on peripheral artery disease (PAD) in hemodialysis patients with dyslipidemia. Therefore, this study aimed to evaluate the effects of eicosapentaenoic acid (EPA) and docosahexaenoic acid (DHA) on the ankle–brachial index (ABI) and remnant-like lipoprotein cholesterol (RLP-C) levels, which are indicators of PAD severity. *Materials and Methods:* Thirty-eight participants (mean age: 73.6 ± 12.7 years) were randomly assigned using stratified block randomization to either conventional therapy alone or conventional therapy supplemented with high-dose EPA/DHA (EPA: 1860 mg; DHA: 1500 mg) for a three-month intervention period. Patients in the conventional therapy alone group who opted to continue were provided with a low-dose EPA/DHA regimen (EPA: 930 mg; DHA: 750 mg) for an additional three months. The baseline and 3-month values for RLP-C, an atherogenic lipid parameter, and the ABI were recorded. *Results:* The results of the 3-month assessments revealed that the mean RLP-C changes were −3.25 ± 3.15 mg/dL and 0.44 ± 2.53 mg/dL in the EPA/DHA and control groups, respectively (*p* < 0.001), whereas the changes in the mean ABI values were 0.07 ± 0.11 and −0.02 ± 0.09 in the EPA/DHA and control groups, respectively (*p* = 0.007). In the EPA/DHA group, a significant negative correlation was found between the changes in RLP-C levels and the ABI (r = −0.475, *p* = 0.04). Additionally, the change in the RLP-C levels independently influenced the change in the ABI in the EPA/DHA group, even after adjusting for age, sex, and statin use (*p* = 0.042). *Conclusions:* Add-on EPA/DHA treatment improved the effectiveness of conventional therapy (such as statin treatment) for improving the ABI in hemodialysis patients with dyslipidemia by lowering RLP-C levels. Therefore, clinicians involved in dialysis should focus on RLP-C when considering residual cardiovascular disease risk in hemodialysis patients and should consider screening patients with elevated levels.

## 1. Introduction

End-stage renal disease requires hemodialysis and is associated with various adverse effects, including an increased risk of major adverse cardiovascular events and mortality [1,2]. The mechanisms underlying the association between end-stage renal disease and major adverse cardiovascular events include highly prevalent atherosclerotic comorbidities, such as coronary artery disease, cerebrovascular disease, and peripheral artery disease (PAD) [2,3]. PAD is common in patients undergoing hemodialysis and is associated with high risks of major adverse cardiovascular events, hospitalization, and reduced health-related quality of life [3]. Moreover, a recent study has indicated that the ankle–brachial index (ABI), which is an indicator of PAD severity, can effectively predict the occurrence of major adverse cardiovascular events in hemodialysis patients [4].

Additionally, PAD in hemodialysis patients has been strongly linked to frailty, impacting life expectancy and quality of life [5]. Patients with PAD often experience social and emotional impairments, affecting their ability to engage in social activities, leisure, and employment [6]. Thus, effective treatments are urgently needed to mitigate these risks. This is particularly crucial given the significant healthcare costs associated with managing PAD in patients with diabetes, estimated at USD 84–380 billion annually in the United States [7].

Notably, a low ABI, even borderline, has been independently associated with lower quality of life in older adults in the Atherosclerosis Risk in Communities Study [8]. Further research is required to determine if specific PAD management strategies can enhance the quality of life in older populations. Although subjective patient outcomes such as pain and fatigue are essential determinants of prognosis, they have not received adequate attention from medical professionals [9], highlighting the need for future solutions that address these outcomes.

Lipid abnormalities, causing cellular oxidative injuries and pro-inflammatory conditions, are prevalent in patients with chronic kidney disease (CKD) [10]. Remnant-like lipoprotein cholesterol (RLP-C) is a potent atherogenic lipid with elevated levels in severe chronic renal failure requiring hemodialysis [11,12]. It independently increases the risk of endothelial dysfunction and coronary artery disease [13]. RLP-C also plays a significant role in atherosclerosis development [14] and represents a target for reducing residual risk after achieving target low-density lipoprotein (LDL) cholesterol levels [15]. However, its effects on hemodialysis patients and its role in PAD still need to be explored [16,17].

An elevated risk of cardiovascular disease (CVD) persists in patients with end-stage renal disease even after receiving statin-based treatment for managing LDL cholesterol [12], indicating the need for alternative therapeutic targets with the potential to further reduce cardiovascular risk. Furthermore, the RLP-C level is a key residual risk factor for future cardiovascular events in patients with stable coronary artery disease whose LDL cholesterol levels are maintained below 70 mg/dL using statins [18]. Omega-3 fatty acids, such as eicosapentaenoic acid (EPA) and docosahexaenoic acid (DHA), are known to reduce RLP-C levels in hemodialysis patients [19] and patients with nephrotic range proteinuria [20], with a significant dose-dependent effect [21]. This reduction in RLP-C may occur through several mechanisms, including the inhibition of hepatic triglyceride production and secretion, the enhanced clearance of triglyceride-rich lipoproteins, and the modulation of gene expression related to lipid metabolism [22]. However, there are limited reports confirming a reduction in RLP-C with EPA/DHA administration in hemodialysis patients. Therefore, the present study aimed to investigate whether high-dose EPA and DHA treatments can improve PAD severity by reducing the RLP-C levels as a novel strategy for managing PAD in hemodialysis patients with dyslipidemia.

## 2. Materials and Methods

### 2.1. Study Design

This open-label randomized pilot study enrolled eligible hemodialysis patients with dyslipidemia, who were randomized using a stratified block randomization method to receive either conventional therapy alone (control group) or conventional therapy with add-on high-dose EPA/DHA treatment (1860 mg of EPA and 1500 mg of DHA; high-dose EPA/DHA group) for 3 months. The conventional therapy used in the present study included administering antiplatelet agents (if necessary), lipid-lowering drugs (mainly statins at appropriate doses), and antihypertensive drugs, as recommended by the Japan Atherosclerosis Society guidelines [23]. Following conventional therapy, some of the patients then continued to receive an add-on treatment of low-dose EPA/DHA (930 mg of EPA and 750 mg of DHA; low-dose EPA/DHA group) for an additional 3 months. The RLP-C levels and ABIs were measured as an atherogenic lipid parameter and an indicator of PAD severity, respectively, and were assessed at baseline and after 3 months.

### 2.2. Participants

Consecutive patients who were admitted to Yokosuka City Hospital between July and November 2015 were recruited. The study was approved by the ethics committee of Yokosuka City Hospital and registered at the University Hospital Medical Information Network (UMIN000017853). All participants provided written informed consent prior to the study enrollment. The inclusion criteria were as follows: patients (1) who underwent hemodialysis with a diagnosis of dyslipidemia, (2) who were aged ≥ 20 years, (3) who did not receive omega-3 fatty acids (including supplements) in the last 6 months, and (4) who provided voluntary informed consent after receiving a full explanation of the study’s purpose and methods. In contrast, the exclusion criteria were as follows: patients (1) with a history of allergy to omega-3 fatty acids, (2) with confirmed or suspected pregnancy, (3) with active bleeding, and (4) who were removed from the study for any reason at the investigators’ discretion.

### 2.3. Sample Size Calculation

Using a clinically valid ABI difference of 0.1 and calculating the sample size with a 10% significance level, 70% power, and a two-tailed test using Student’s *t*-test against a standard deviation of 0.17 for the ABI in our dialysis patients, the total sample size was calculated as 56 patients. To take into account potential dropouts from the study, the final sample size was set at 60 patients.

### 2.4. Demographic and Clinical Assessments

Trained nurses collected blood samples (fasting state) from each patient for laboratory testing to measure serum levels of LDL cholesterol, high-density lipoprotein (HDL) cholesterol, triglycerides, and RLP-C. The LDL cholesterol level was measured by an enzymatically direct measure method using the kit from DENKA SEIKEN, Inc. (Tokyo, Japan). The RLP-C levels were measured enzymatically using a RemL-C kit from MINARIS Medical, Inc. (Tokyo, Japan). The RemL-C assay utilizes surfactant POE-POB block copolymer and phospholipase D, which can selectively solubilize and degrade TG-rich remnant lipoproteins, VLDL remnants, and chylomicron remnants. Then, the released cholesterol was measured enzymatically. POE-POB selectively binds to very-low-density lipoprotein remnants and intermediate-density lipoprotein particles, and the phospholipase D addition supports the reactivity towards chylomicron remnants. This RemL-C assay was performed with an autoanalyzer (Hitachi 7600 automated analyzer, Tokyo, Japan). Dyslipidemia was defined as serum LDL cholesterol, HDL cholesterol, and triglyceride concentrations of ≥140 mg/dL, <40 mg/dL, and ≥150 mg/dL, respectively, or treatment with lipid-lowering drugs, such as a statin. A history of diabetes was defined as a history of physician-diagnosed diabetes, the use of medication for diabetes, a fasting blood glucose level of ≥126 mg/dL, or a non-fasting blood glucose level of ≥200 mg/dL, as previously described [24]. Patients with diabetes received appropriate treatments, including the use of antidiabetic drugs, in accordance with the Japanese Clinical Practice Guideline for Diabetes to achieve a hemoglobin A1c level of <7%.

Each participant’s ABI was measured using the Vascular Screening System VaSera VS-2000 (Fukuda Denshi, Tokyo, Japan). Firstly, each patient was asked to rest for 10 min. Following this, their systolic blood pressure was measured at each arm and leg while they were placed in the supine position with straight arms and legs. The ABI value was calculated as the ratio of the ankle systolic blood pressure divided by the brachial systolic blood pressure, and the lowest value from the right or left side was recorded and used in the analyses.

### 2.5. Statistical Analysis

All analyses were performed using the IBM SPSS software version 22.0 (IBM Corp., Armonk, NY, USA). The differences between the EPA/DHA and control groups were evaluated using the Student’s *t*-test, Mann–Whitney U test, or chi-squared test. A sample size calculation was performed using power analysis. Pearson’s correlation analysis and multiple linear regression analysis were used to evaluate the relationships between the changes in the RLP-C level and the ABI. All data are expressed as the mean ± standard deviation or a number (percentage). A *p*-value of <0.05 (two-sided) was considered significant.

## 3. Results

**Figure 1:**
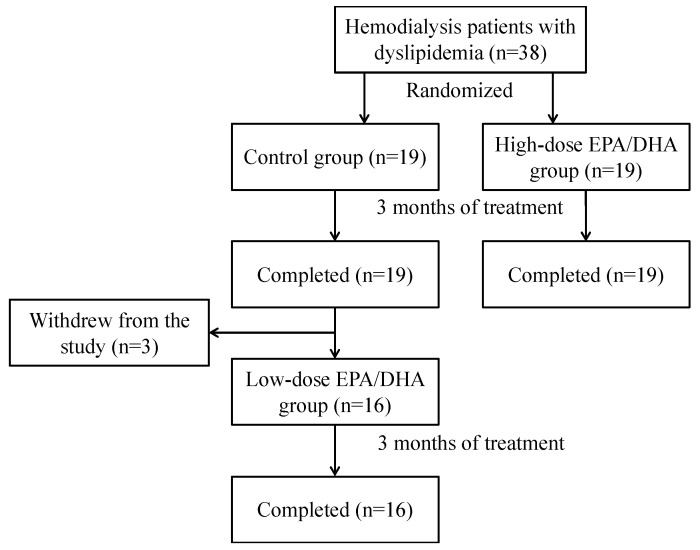
Flowchart of the patient selection process. Following the 3-month evaluation, 16 out of the 19 patients in the control group continued to receive treatment with low-dose EPA/DHA for an additional 3 months. DHA: docosahexaenoic acid; EPA: eicosapentaenoic acid.

**Figure 2:**
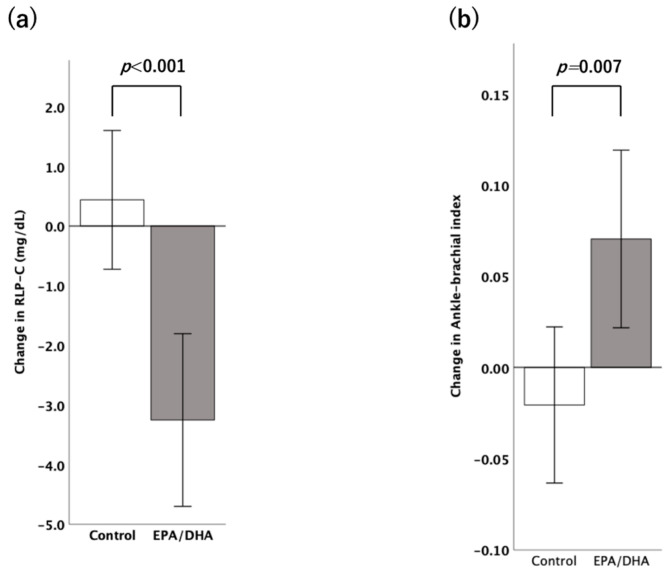
Changes in the (**a**) RLP-C levels and (**b**) Ankle-brachial index in the control and EPA/DHA groups after 3 months of treatment. Data are expressed as the mean ± standard error of the mean. The control group received statin treatment alone, whereas the high-dose EPA/DHA group received EPA and DHA along with statins. DHA: docosahexaenoic acid; EPA: eicosapentaenoic acid; RLP-C: remnant-like lipoprotein cholesterol.

This study included 38 eligible hemodialysis patients with dyslipidemia (control group: *n* = 19 and high-dose EPA/DHA group: *n* = 19). Figure 1 shows a flowchart of the patient selection process, with all participants in the control and high-dose EPA/DHA groups completing the 3-month evaluation. After the 3-month evaluation, 16 of the 19 patients in the control group continued treatment with low-dose EPA/DHA for an additional 3 months. The patients’ characteristics are presented in Table 1. Of the 38 eligible patients, 44.7% were treated with statins, and the LDL cholesterol control status was 55.3% below 70 mg/dL and 34.2% below 55 mg/dL. No significant differences were found between the control and high-dose EPA/DHA groups, even in terms of statin use (all statins were strong statins). Five patients in the control group (26.3%) and three in the EPA/DHA high-dose group (15.8%) had abnormal ABIs (<0.9 and >1.4); two (10.5%) and eight (42.1%) patients, respectively, had abnormal TG levels (>150 mg/dL); and four (21.1%) and nine (47.4%) patients, respectively, had abnormal RLP-C levels (>7.5 mg/dL). After the 3-month intervention, the mean RLP-C changes were −3.25 ± 3.15 mg/dL and 0.44 ± 2.53 mg/dL in the high-dose EPA/DHA and control groups, respectively (inter-group difference in mean RLP-C changes: 3.69 mg/dL; *p* < 0.001) (Figure 2a). Furthermore, compared to the EPA/DHA low-dose group, the EPA/DHA high-dose group showed a marked and volume-dependent decrease in RLP-C (−0.37 ± 3.50 mg/dL and −3.25 ± 3.15 mg/dL, *p* = 0.015). The mean ABI changes were 0.07 ± 0.11 and −0.02 ± 0.09 in the high-dose EPA/DHA and control groups, respectively (inter-group difference in mean ABI changes: 0.09; *p* = 0.007; Figure 2b).

Table 2 shows the changes in lipid and inflammatory parameters after 3 months in the control and high-dose EPA/DHA groups. TG and RLP-C were predominantly reduced in the high-dose EPA/DHA group compared to the control group (5.8 ± 34.6 mg/dL in the control group versus −41.0 ± 45.2 mg/dL in the high-dose EPA/DHA group, *p* < 0.001; 0.44 ± 2.53 mg/dL in the control group versus −3.25 ± 3.15 mg/dL in the high-dose EPA/DHA group, *p* < 0.001).

**Figure 3:**
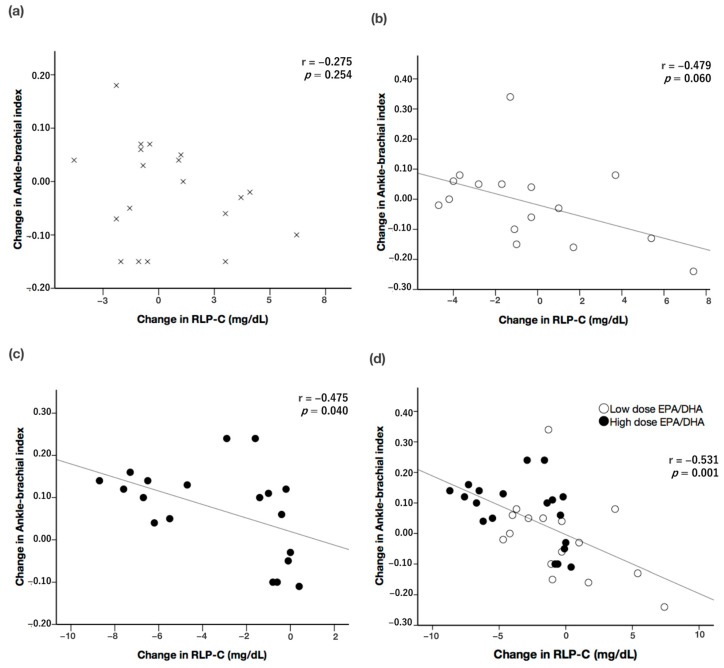
Correlation between the changes in RLP-C levels and ABI values in the (**a**) control group, in “x” (**b**) low-dose EPA/DHA group, in white circles (**c**) high-dose EPA/DHA group, in black circles and (**d**) low- and high-dose EPA/DHA groups combined in white and black circles, respectively. ABI: ankle–brachial index; DHA: docosahexaenoic acid; EPA: eicosapentaenoic acid; n.s.: not significant; RLP-C: remnant-like lipoprotein cholesterol.

In the control group, no significant correlation was observed between the changes in RLP-C levels and ABI values (r = −0.275, *p* = 0.254; Figure 3a). In the low-dose EPA/DHA group, a negative correlation was observed between the changes in RLP-C levels and the ABI (r = −0.479, *p* = 0.06; Figure 3b). In the high-dose EPA/DHA group, a significant negative correlation was observed between the changes in RLP-C levels and ABI values (r = −0.475, *p* = 0.04; Figure 3c). Furthermore, when the low- and high-dose EPA/DHA groups were combined, a significant negative correlation was found between the changes in RLP-C levels and ABI values (r = −0.531, *p* = 0.001; Figure 3d). The multiple regression analysis revealed that the change in RLP-C levels was an independent determinant of the change in the ABI (Table 3) in Model 2 (adjusted for age, sex, duration of hemodialysis, history of diabetes, and statin use; β = −0.617, *p* < 0.001) and Model 3 (adjusted for the covariates in Model 2 plus the baseline SBP, TG, LDL-C, HDL-C, EPA, and DHA concentrations; β = −0.653, *p* < 0.001) in the combined low- and high-dose EPA/DHA groups. Of the 35 patients receiving EPA/DHA, the correlation between the ABI and the RLP-C level in the 15 patients receiving statins was r = −0.454, *p* = 0.089, while the correlation between ABI and RLP-C in the 20 patients not receiving statins was r = −0.659, *p* = 0.002. No serious adverse events occurred in any patient group, including cardiovascular disease or significant bleeding.

## 4. Discussion

The present study evaluated hemodialysis patients with dyslipidemia and demonstrated that high-dose EPA/DHA treatment provided an added benefit when combined with conventional therapy for PAD. Furthermore, an improvement in PAD severity was correlated with a reduction in RLP-C level, and this relationship was independent of statin use. These findings are consistent with those of a previous study that reported that high-dose EPA treatment prevented the occurrence of cardiovascular events [25], whereas low-dose EPA demonstrated no significant effect in preventing these events [25,26]. This discrepancy may be related to the dose-dependent effects of omega-3 fatty acids (such as EPA and DHA) on atherogenic lipids [21], suggesting that high doses of EPA and DHA are required to adequately reduce the level of atherogenic lipids. The relationship between hemodialysis and atherosclerosis is complicated; however, atherogenic lipoproteins accumulate in the plasma of hemodialysis patients, leading to the development of atherosclerosis [27]. Furthermore, RLP-C is a strong atherogenic lipid, and its level becomes elevated in patients with severe chronic renal failure requiring hemodialysis [11,12].

Notably, treatment with high-dose EPA/DHA helped reduce PAD severity with or without appropriate statin use, with the baseline mean LDL cholesterol level (69.0 mg/dL) comparable to that reported in a previous study (75.0 mg/dL) [25]. High RLP-C levels increase the incidence of cardiovascular events in patients with stable cardiovascular disease and statin-treated LDL-C levels below 70 mg/dL [18]; therefore, high doses of EPA/DHA could potentially also reduce the incidence of such events in these patients. In this study, 44.7% of the 38 eligible patients were treated with statins, 55.3% had LDL cholesterol levels below 70 mg/dL, and 34.2% had controlled LDL cholesterol levels below 55 mg/dL, so LDL cholesterol was not necessarily adequately treated. Therefore, it is not clear whether high doses of EPA/DHA are effective in patients who are adequately treated with statins and whose LDL cholesterol is tightly controlled. Furthermore, in this study, among the 35 patients receiving EPA/DHA, the correlation between the ABI and RLP-C level in the 15 patients taking statins was r = −0.454, *p* = 0.089, while the correlation between the ABI and RLP-C level in the 20 patients not receiving statins was r = −0.659, *p* = 0.002. These results suggest that a reduction in RLP-C and an improvement in the ABI may be associated, regardless of statin administration. However, it is important to note that the interpretation of the regression analysis results in this study is limited by the small number of cases. Therefore, further research is required to confirm this association. A recent meta-analysis [12] also indicated that, despite statin treatment, 13% of the patients with stage 3 chronic kidney disease had experienced major cardiovascular events, while 22% with stage 5 CKD had experienced cardiovascular events. Therefore, the residual risk of cardiovascular events continues to rise in the late stages of chronic kidney disease. This finding may partly be explained by the increase in atherogenic lipoproteins, such as RLP-C, as renal failure progresses [10]. Furthermore, an observational study that followed hemodialysis patients for 10 years reported that the risk of cardiovascular events increased as cholesterol levels increased. This finding indicates that an intensified cholesterol-lowering treatment may be necessary to reduce the risk of CVD [28]. Of interest, in this study, 3 months of high-dose EPA/DHA reduced RLP-C by approximately 46%, whereas a randomized controlled trial of cilostazol for 6 months in patients with intermittent claudication (n = 56) reduced RLP-C by approximately 20% [29]. However, this comparison needs to be specifically examined further in a randomized controlled trial.

Interestingly, the correlation between the reduction in RLP-C and the improvement in ABI was independently associated with a history of dialysis, the presence of diabetes, and baseline EPA and DHA concentrations. This may suggest that in patients with higher RLP-C, PAD may improve regardless of the duration of time since the induction of dialysis, and that improvement in PAD may be observed regardless of the presence or absence of diabetes mellitus. The risk of cardiovascular disease in diabetic patients remains high even with the use of statin therapy to lower LDL cholesterol. Diabetic nephropathy is the most common cause of dialysis induction, and high blood glucose levels increase triglycerides as the liver is more likely to produce triglycerides from excess sugar and insulin [30]. In the present study, EPA/DHA treatment improved the ABI with a decrease in RLP-C, irrespective of the presence or absence of diabetes. However, due to the small number of cases, future validation in large-scale clinical trials is required.

RLP-C (produced by lipolysis from very-low-density lipoproteins and chylomicrons) is thought to contribute to this residual risk, although the factors mediating such a risk are not completely understood [31]. It has long been known that statins, in combination with EPA/DHA, contribute to RLP-C reduction [20], and the results of this study, in which EPA/DHA significantly reduced RLP-C, support this finding. In addition, the results suggest that a reduction in RLP-C may improve the ABI, regardless of baseline EPA or DHA excess or deficiency. The ability to improve the ABI regardless of EPA and DHA concentrations suggests that this medication could be easy to use in everyday clinical practice. These factors suggest that EPA and DHA are potential therapeutic agents for reducing cardiovascular risk in patients with end-stage renal disease who are already receiving adequate statin-based treatment because of their effectiveness in reducing RLP-C levels. However, the improvement in the ABI cannot be explained solely by the decrease in RLP-C caused by EPA/DHA, and a combination of factors such as anti-inflammatory, anticoagulant, and lipid-lowering effects may be responsible for the improvement in the ABI. Therefore, further clinical studies are needed.

A previous systematic review found that omega-3 fatty acids improved vascular access outcomes [32]; however, the trials included in the systematic review were of poor quality, and many used low doses of omega-3 fatty acids (especially DHA). Nevertheless, a previous study reported that there is an association between low DHA and increased PAD severity [33]; therefore, we believe that our finding that the use of high doses of DHA decreases the severity of PAD is partially supported by this study.

Notably, a large cohort study showed that omega-3 fatty acids and statin reduced the risk of all-cause death, composite outcomes of endovascular treatment, and amputation in a dose-dependent manner in hemodialysis patients with dyslipidemia [34]. Furthermore, omega-3 fatty acids were associated with the development of cardiovascular disease and myocardial infarction in a meta-analysis including 40 studies and 135,267 patients. The association was found to be dose-dependent and strong [35], supporting the findings of our study. These findings suggest that enhancing the treatment of dyslipidemia, such as by increasing the dosage, may be important in improving the outcomes of hemodialysis patients with dyslipidemia.

In alignment with the current European Society of Cardiology and European Atherosclerosis Society guidelines [36], the continuation of statins, ezetimibe, or a statin/ezetimibe combination is recommended for patients already on these medications at the initiation of dialysis, especially those with a history of CVD. Conversely, the guidelines suggest that in patients with dialysis-dependent CKD who are free of CVD, the commencement of statin therapy is not recommended. Thus, in hemodialysis patients with limited therapeutic options for dyslipidemia, EPA/DHA may be a potential choice for preventing or treating PAD. Further investigation is warranted to explore this possibility.

Clinical indicators for disease severity, such as low ABI scores, are associated with reduced health-related quality of life; however, patients with the same ABI can have widely varying symptoms, functioning, and health-related quality of life [8]. Since quality-of-life-related indicators were not measured in this study, future studies should evaluate these indicators. A low ABI (even in the borderline range) is independently associated with a lower quality of life in community-dwelling older adults, especially from a physical perspective [8]. Therefore, improving the ABI may be important for improving the quality of life of older hemodialysis patients.

## 5. Limitation

The present study has several limitations.

First, the open-label pilot study design, small sample size, and short follow-up duration might have led to biased and heterogenous findings. For example, this study originally planned to include 60 patients as the calculated required sample size; however, only 38 patients were enrolled, as the number of patients was lower than expected. Therefore, a large-scale, double-blind, randomized controlled trial with a long follow-up duration is needed to validate our findings.

Second, we only considered PAD improvement based on the ABI scores; however, it would be useful to confirm the results using other PAD screening methods, such as the 6-min walking test and pain-free walking distance, or other modalities for evaluating PAD, such as flow-mediated dilation, skin perfusion pressure, and brachial-ankle pulse wave velocity [37].

Third, we did not maintain a record of the usage of the remaining medications in this study. However, since the patients were verbally asked if they consumed residual medications and no significant residual medication use was noted, we do not believe that medication compliance was affected significantly.

Fourth, another limitation is that we are not able to rigorously assess the type of dyslipidemia.

Finally, the multiple regression analysis results showed that the RLP-C-lowering effect was independent of statin use; however, the statin dose was not standardized. Therefore, a fixed statin dose should be used in future studies to further validate this finding.

## 6. Future Research Directions

The importance of patients’ subjective outcomes has recently been discussed [9], and the study could have been made even better by also measuring the effects of improvements in patients’ quality of life and well-being. Third, large cohort studies have found that a high intake of marine n-3 polyunsaturated fatty acids does not reduce the risk of coronary heart disease or ischemic stroke [38]; therefore, it is necessary to examine the dietary intake of polyunsaturated fatty acids and omega-3-fatty acids, as different preparations do not always have consistent results. To assess further studies, we should focus on quality-of-life-related indicators. Additionally, a large-scale, double-blind, randomized controlled trial with a long follow-up duration is required.

## 7. Conclusions

In hemodialysis patients with dyslipidemia receiving conventional statin-based therapy, the add-on high-dose EPA/DHA treatment improved their ABI. Therefore, the dose-dependent effects of EPA/DHA on RLP-C levels may be an important factor in PAD improvement. Clinicians involved in dialysis care might focus on RLP-C, an atherogenic lipid, when considering residual cardiovascular disease risk in hemodialysis patients, and it may also be worthwhile to consider specifically screening patients with elevated RLP-C levels. Moreover, EPA/DHA may be a good indication for improving the ABI in hemodialysis patients with elevated RLP-C. The reduction in RLP-C levels by EPA/DHA has the potential to improve patients’ quality of life and contribute to a reduction in medical costs, providing a multitude of benefits.

## Figures and Tables

**Table 1:** Participant characteristics.

	Control Group *n* = 19	High-Dose EPA/DHA Group *n* = 19	*p*-Value
Age, years	73.6 ± 12.7	66.3 ± 11.2	0.068
Female sex, *n* (%)	7 (36.8)	4 (21.1)	0.476
Systolic blood pressure, mmHg	147.6 ± 16.4	157.4 ± 22.9	0.139
Diastolic blood pressure, mmHg	78.7 ± 10.5	80.2 ± 11.6	0.685
LDL cholesterol level, mg/dL	60.0 (51.0)	73.5 (37.5)	0.760
HDL cholesterol level, mg/dL	48.5 (18.8)	47.0 (27.5)	0.963
Triglyceride level, mg/dL	101.5 (43.8)	113.5 (119.8)	0.785
RLP cholesterol level, mg/dL	4.65 (3.27)	5.65 (8.73)	0.208
Non-HDL cholesterol level, mg/dL	78.5 (42.5)	91.5 (44.0)	0.640
Apolipoprotein B level, mg/dL	59.5 (18.5)	67.0 (18.5)	0.330
CRP level, mg/dL	0.10 (0.23)	0.07 (0.14)	0.453
Ankle–brachial index	1.09 ± 0.21	1.11 ± 0.14	0.727
Duration of hemodialysis, months	49.0 (43.8)	57.0 (116.0)	0.865
History of diabetes, *n* (%)	10 (52.6)	11 (57.9)	1.000
History of CVD, *n* (%)	6 (31.6)	2 (10.5)	0.741
History of PAD, *n* (%)	4 (21.1)	2 (10.5)	0.660
Use of statins, *n* (%)	9 (47.4)	8 (42.1)	1.000
Use of statins before initiation of hemodialysis, *n* (%)	12 (63.2)	11 (57.9)	1.000
Use of ezetimibe, *n* (%)	4 (21.1)	3 (15.8)	1.000

Data are expressed as the mean ± standard deviation, median (interquartile range), or number (percentage). DHA: docosahexaenoic acid; EPA: eicosapentaenoic acid; HDL: high-density lipoprotein; LDL: low-density lipoprotein; RLP: remnant-like lipoprotein; CRP: c-reactive protein; CVD: cardiovascular disease; PAD: peripheral artery disease. The differences between the EPA/DHA and control groups were evaluated using the Student’s *t*-test, Mann–Whitney U test, or chi-squared test. A *p*-value of <0.05 (two-sided) was considered significant.

**Table 2:** Changes in lipid and inflammatory profiles after 3 months.

	Control Group *n* = 19	High-Dose EPA/DHA Group *n* = 19	*p*-Value
LDL cholesterol level, mg/dL	−3.7 ± 12.2	−4.3 ± 13.5	0.880
HDL cholesterol level, mg/dL	3.8 ± 14.6	2.5 ± 6.9	0.725
Triglyceride level, mg/dL	5.8 ± 34.6	−41.0 ± 45.2	<0.001
RLP-cholesterol level, mg/dL	0.44 ± 2.53	−3.25 ± 3.15	<0.001
Non-HDL cholesterol level, mg/dL	−4.5 ± 20.1	−14.8 ± 33.6	0.260
Apolipoprotein B level, mg/dL	−1.95 ± 9.26	−1.68 ± 9.39	0.931
CRP level, mg/dL	0.22 ± 0.67	−0.02 ± 0.09	0.143

Data are expressed as the mean ± standard deviation. DHA: docosahexaenoic acid, EPA: eicosapentaenoic acid, HDL: high-density lipoprotein; LDL: low-density lipoprotein; RLP: remnant-like lipoprotein; CRP: c-reactive protein. The differences between the EPA/DHA and control groups were evaluated using the Student’s *t*-test. A *p*-value of <0.05 (two-sided) was considered significant.

**Table 3:** Multiple regression analysis of the association between the changes in RLP-C levels and the ABI.

	Β	*p*-Value
Model 1	−0.555	0.001
Model 2	−0.617	<0.001
Model 3	−0.653	<0.001

Model 1: no adjustment. Model 2: adjusted for age, sex, duration of hemodialysis, history of diabetes, and statin use. Model 3: adjusted for the variables in Model 2 plus the baseline SBP, TG, LDL-C, HDL-C, EPA, and DHA levels.

## Data Availability

Data sharing is not possible based on the description of the research protocol approved by the ethics committee.
